# The Biosurfactant *β*-Aescin: A Review on the Physico-Chemical Properties and Its Interaction with Lipid Model Membranes and Langmuir Monolayers

**DOI:** 10.3390/molecules25010117

**Published:** 2019-12-27

**Authors:** Ramsia Geisler, Carina Dargel, Thomas Hellweg

**Affiliations:** 1 Physical and Biophysical Chemistry, Bielefeld University, 33615 Bielefeld, Germany; ramsia@geisler.digital (R.G.); carina.dargel@uni-bielefeld.de (C.D.); 2 Soft Matter at Interfaces, Technical University of Darmstadt, 64289 Darmstadt, Germany

**Keywords:** saponin, *β*-aescin, *β*-escin, lipid membrane, Langmuir monolayers, air–water interface, MD simulation, critical micelle concentration cmc, *Aesculus hippocastanum*

## Abstract

This review discusses recent progress in physicochemical understanding of the action of the saponin β-aescin (also called β-escin), the biologically active component in the seeds of the horse chestnut tree *Aesculus hippocastanum*. β-Aescin is used in pharmacological and cosmetic applications showing strong surface activity. In this review, we outline the most important findings describing the behavior of β-aescin in solution (e.g., critical micelle concentration (cmc) and micelle shape) and special physicochemical properties of adsorbed β-aescin monolayers at the air–water and oil–water interface. Such monolayers were found to posses very special viscoelastic properties. The presentation of the experimental findings is complemented by discussing recent molecular dynamics simulations. These simulations do not only quantify the predominant interactions in adsorbed monolayers but also highlight the different behavior of neutral and ionized β-aescin molecules. The review concludes on the interaction of β-aescin with phospholipid model membranes in the form of bilayers and Langmuir monolayers. The interaction of β-aescin with lipid bilayers was found to strongly depend on its cmc. At concentrations below the cmc, membrane parameters are modified whereas above the cmc, complete solubilization of the bilayers occurs, depending on lipid phase state and concentration. In the presence of gel-phase phospholipids, discoidal bicelles form; these are tunable in size by composition. The phase behavior of β-aescin with lipid membranes can also be modified by addition of other molecules such as cholesterol or drug molecules. The lipid phase state also determines the penetration rate of β-aescin molecules into lipid monolayers. The strongest interaction was always found in the presence of gel-phase phospholipid molecules.

## 1. Introduction

β-Aescin or β-escin belongs to the vast family of natural surfactants, the so-called saponins [1]. All saponins follow very similar structural motifs. They consist of an aglycone (hydrophobic part of the molecule) which is linked to one or several oligosaccharide chains. Saponins are found in hundreds of plants and a lot of them have interesting pharmacological activities which have already been used in ancient times for the treatment of various diseases. The very characteristic amphiphilic nature of the saponins leads to diverse applications in, among others, industry and pharmacy [2,3,4,5,6,7,8].

**Figure 1:**
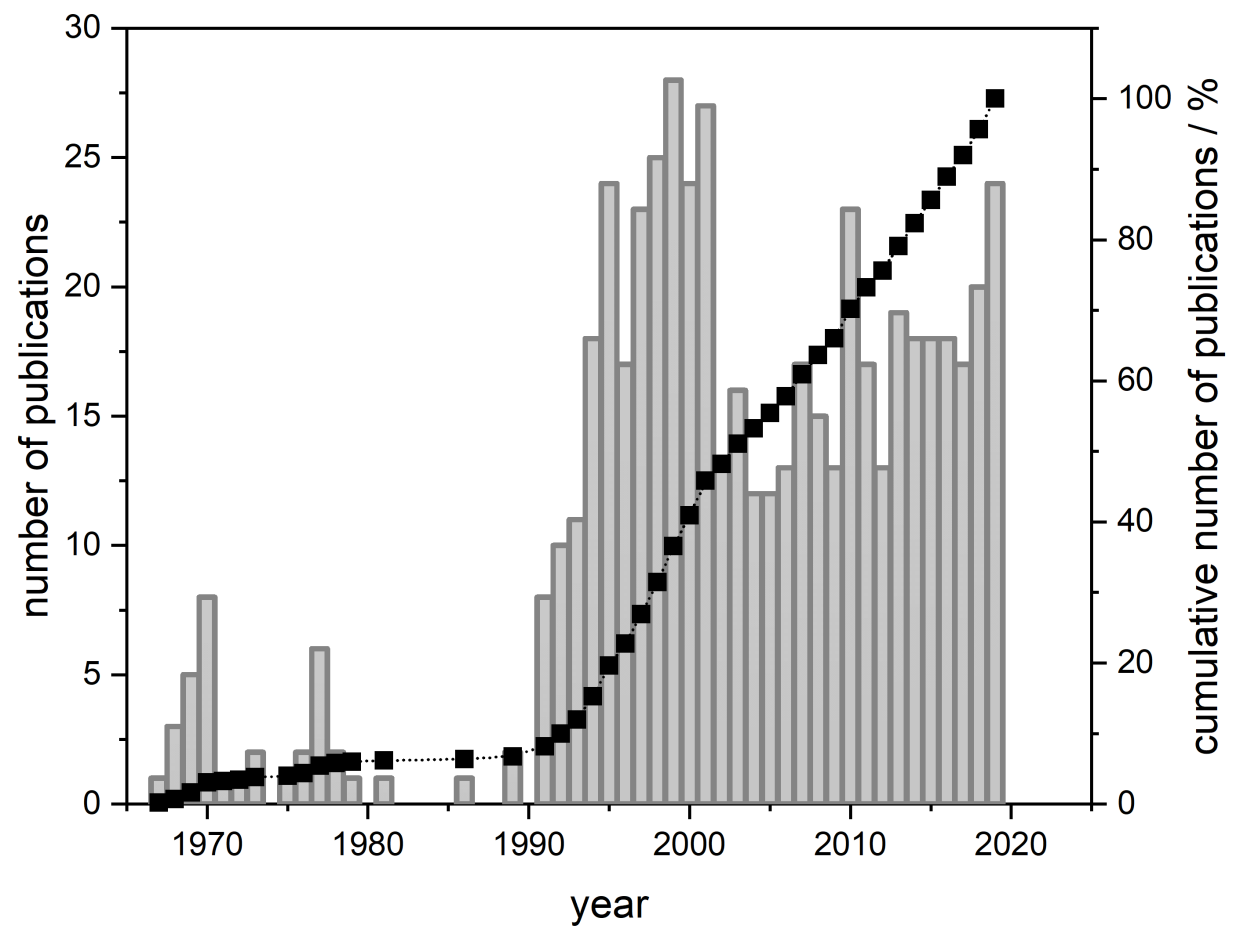
Number of publications related to the saponin β-aescin in the years 1967 to 2019. The data is obtained from a search in the ”The Web of Science Core Collection” database under the keyword (a)escin.

A simple search in the ”The Web of Science Core Collection” database yields 550 hits for β-aescin at the moment (November 2019, compare Figure 1). More than one third of these works was published in the last 10 years. This shows the continuous interest in this natural surfactant molecule produced by the horse chestnut tree *Aesculus hippocastanum*. However, most of the published works are pharmacological studies which have been recently reviewed by Xinxin Zhang et al. [9]. β-Aescin has been proven to be safe and very tolerable [10,11]. It is, e.g., successfully used in the treatment of the post thrombotic syndrome [12,13], chronic venous insufficiency [10,14,15], shows promising effects in the treatment of arthritis [16,17], and seems to have potential as an anti-cancer drug [18,19]. Especially remarkable is β-aescins action against glioblastoma-initiating cells (GIC) which have the ability to initiate glioblastoma growth [20]. Glioblastoma multiform is the most abundant primary tumor of the human nervous system. β-Aescin was more efficient in diminishing GIC growth compared to present clinically used drugs. Moreover, as other saponins, it shows a hemolytic effect and seems to interact with non-steroidal anti-inflammatory drugs inside lipid membranes [21]. Vinarov et al. have shown that β-aescin might also help to solubilize hydrophobic drugs [22]. Also most of the available reviews mainly treat the biological activity of the β-aescin molecule. The most recent one was published by Luca Galleli in September 2019 [23].

The physical properties of β-aescin are less investigated and its interaction with biological membranes on a molecular level, which is the basis of several of its effects, is only sparsely studied. Hence, in the present focus review, we will try to summarize recent progress in this area.

The review is organized as follows. Section 2 will introduce the chemical structure of β-aescin. In Section 3 the micelle formation properties of β-aescin will be investigated. Afterwards, in Section 4, the behavior of β-aescin at interfaces is focused upon. In Section 5, we will review and discuss the interaction of β-aescin with bare lipid membranes, followed by Section 6 which describes synergistic effects between other membrane active compounds like e.g., cholesterol or drugs. In Section 7, the interaction with Langmuir monolayers is summarized. The focus review will end with a conclusion (Section 8) also comprising remarks about future perspectives in β-aescin research.

## 2. The Saponin β-Aescin: Chemical Structure and Specifications

Saponins generally share a similar structure, which is built by an either triterpenic or steroidic hydrophobic backbone, to which a different number of sugar chains is attached [1,2,3,4,5,6,7,8]. Aescin itself obtained from the seeds of the horse chestnut tree is not a pure compound but rather a complex mixture of about 30 different molecules [7,10,24,25]. This mixture is based on two different hydrophobic, triterpenic backbones, namely, protoaescigenin and barringtogenol C [2]. Both backbones differ only at the C-4 position which carries a methyl group in the case of barringtogenol C and a methoxy group in case of protoaescigenin [7]. In all cases, a trisaccharide is attached to the C-3 position of the backbone. Thereby, a glucuronic acid acts as linker for glucose, xylose or galactose molecules [7]. Hydrolysis experiments showed that the main fraction consists of only glucose and glucuronic acid [24].

**Figure 2:**
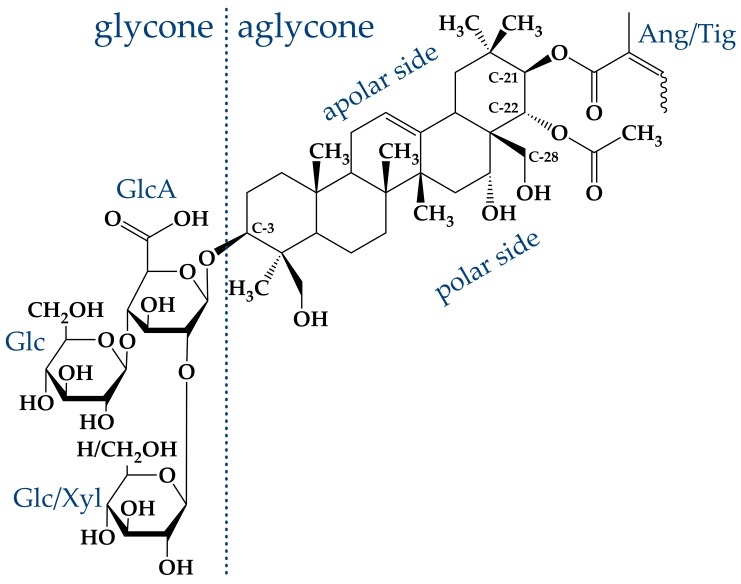
Molecular structure of β-aescin [29]. The amphiphilic structure of β-aescin consists of a hydrophobic, aglyconic part and a hydrophilic, glyconic part. The glyconic part contains glucuronic acid (GlcA), and glucose (Glc) (or xylose (Xyl)). However, in addition to the classical surfactant-like polarity, also within the aglyconic part polar groups are attached one-sided to the triterpene. This gives the aglyconic backbone a slightly polar and a slightly apolar side (Used abbreviations: Ang → angelic acid, Tig → tiglic acid).

The main compound in this “aescin mixture”, which is present in a share of ≈60%, consists of a protoaescigenin backbone which is esterified at the C-22 position with acetic acid and with angelic/tiglic acid at C-21 [6,7,10,26]. This fraction is called β-aescin and the most abundant structures of this mixture are shown in Figure 2. One of the two glucose molecules attached to the glucuronic acid can be substituted by xylose [6,24,27]. Besides β-aescin, two other fractions are identified in the aescin mixture. These are called α- and crypto-aescin, whereby α-aescin is a mixture of crypto- and β-aescin [7,10,28]. β- and crypto-aescin differ in the position of the acetyl group in the backbone [15,28]. Whereas for crypto-aescin this group is located at C-28, it can be found at C-22 for β-aescin. Both forms can be distinguished by the solubility in water, the melting point and the hemolytic index [15]. In comparison to α-aescin, β-aescin shows a much higher pharmacological activity and β-aescin is the main active component in the pharmaceutically used horse chestnut tree extract [10,25].

According to the literature, the amount of the general saponin mixture in the horse chestnut tree seeds is ≈3–10%, whereby the amount of the aescin mixture is ≈2–3% [2,7,10,28,30]. In conventional extraction, saponins are dissolved in a solvent [4]. In the case of aescin, the horse chestnut seeds are first dried and ground up to a powder. This powder is afterwards treated with ≈60% v/v ethanol (or methanol) to dissolve the aescin fraction [31,32]. By e.g., addition of acid and therewith lowering of the pH value, the less soluble protonated form of β-aescin sediments and can be separated afterwards from the α-aescin fraction and other compounds [32]. In 1991, E. Horvath proposed a different approach, which does not require alcohol for extraction and which results in better yields of β-aescin. β-Aescin was extracted from fresh, frozen and ground seeds by the usage of only water as solvent. The pure substance β-aescin is obtained by acidification and recrystallization. A separation of both forms is not necessary, because an isomerization of the β-form to α-aescin induced by drying the seeds is avoided [32].

β-Aescin is commercially available (CAS: 6805-41-0) and can e.g., be purchased from Merck/Sigma-Aldrich Company in purity of >95 %. The structures present in this mixture were determined by combination of analytical reversed-phase high performance liquid chromatography (RP-HPLC) and the analysis techniques mass spectrometry and ^1^H-NMR spectroscopy [29]. Two separable fractions with an exact mass of 1130.5 g/mol were obtained. ^1^H-NMR spectroscopy identified both fractions as cis-trans-isomers with either tiglic or angelic acid attached to the C-21 carbon atom of the triterpenic backbone [29,33,34]. The presence of xylose, which would lead to a different molar mass, was not proven. Hence, these structures correspond to β-aescin. The solubility of the compound in an aqueous solution is limited, because especially the acidic form of the molecule shows a bad solubility [32]. The solubility of β-aescin can be significantly increased by increase of pH value (and the usage of buffer). The carboxylic group located in the glycone of β-aescin (Figure 2) induces intrinsic pH-dependence of the β-aescin form. Dargel et al. [29] recently determined the $pK_{a}$ value of β-aescin in water to be 4.7 ± 0.2. Therefore, it is assumed that β-aescin exists in its neutral form below and in a deprotonated ionized form above a pH value of 4.7. Deprotonation leads to strongly enhanced solubility of β-aescin in water. Moreover, β-aescin is soluble in alcohols such as ethanol and methanol, which are also used in the extraction procedure [31,32].

## 3. Micelle Formation

The amphiphilic nature of β-aescin causes formation of micelles in aqueous solution at a critical micelle concentration (cmc). Values for the cmc of β-aescin have been determined by different groups under different conditions such as varying buffer and pH value. Determination was thereby mainly performed by tensiometry (T) or measurement of the autofluorescence (AF) of β-aescin. The reported values are summarized in Table 1, indicating the pH, solvent, temperature and method used to determine the cmc.

The cmc-values are mostly consistent but, however, show a slight dependence on pH value. Slightly smaller values were obtained at a lower pH value and this observation might be explained by the increased charge of the β-aescin due to deprotonation at higher pH values. β-Aescin molecules were found to exhibit peculiar viscoelastic properties at e.g., the β-aescin–water interface [38,40,41]. These works demonstrated that cmc and pH value are important parameters leading to modified structural and elastic properties of e.g., an adsorbed β-aescin monolayer at the water–air interface. This will be discussed in more detail in Section 4. Also, the state of protonation was found to modify the inter-molecular interactions and thus the attractive and repulsive forces between β-aescin molecules [42,43].

**Figure 3:**
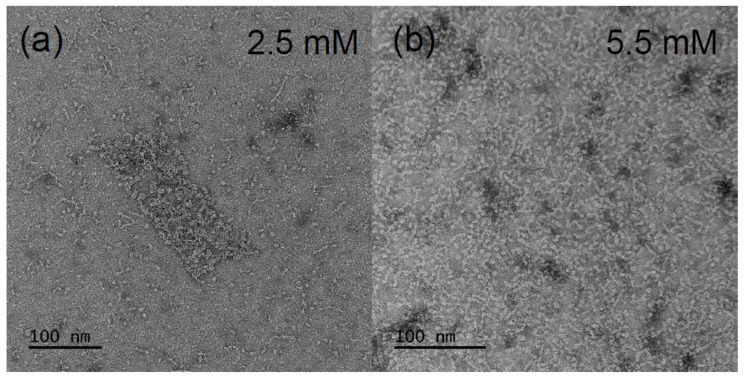
TEM images of dry and stained β-aescin micelles. The concentrations of the solutions are (**a**) 2.5 mM and (**b**) 5.5 mM and were prepared at 6 ${}^{\circ }$C. Reproduced with permission from Dargel et al. [29], Colloids Interfaces; published by MDPI, 2019.

Besides the numerous reports on the cmc-values of β-aescin, there are only two works elucidating the actual structure of β-aescin micelles in solution [29,39]. De Groot et al. [39] report on rather flat and spherically shaped β-aescin micelles in pure water and ambient temperature (Figure 13A) at unspecified pH. In this study, the micelle shape was visualized by transmission electron microscopy (TEM) and an average length/diameter of the β-aescin micelles of 200 Å was obtained. Another work by Dargel et al. [29] characterizes the β-aescin structures in dependence of β-aescin concentration ranging from 1.7–9.5 mM at temperatures of 10 ${}^{\circ }$C and 40 ${}^{\circ }$C. The analysis was conducted by TEM and small-angle X-ray scattering (SAXS) experiments and clearly shows that micellar shape varies with temperature whereas the concentration only plays a marginal role above the cmc. Figure 3 shows TEM images at different concentrations and low temperature (6 ${}^{\circ }$C). The contour of the micelles suggests that they exhibit a rather cylindrical shape with a cross-sectional diameter of 25–30 Å and a length of 70–150 Å. From a closer look, it becomes visible that the rods contain smaller segments which could be stacked smaller discoidal micelles. However, by the SAXS results, a temperature-dependent behavior was evidenced. Whereas, the cylindrical shape could be confirmed at low temperature of 10 ${}^{\circ }$C, ellipsoidal structures form at 40 ${}^{\circ }$C. The rods show a cross-sectional diameter ranging from 16.8 Å to 18.5 Å and a length in the range between 74 Å and 90 Å, which slightly decreased with increasing β-aescin concentration. The equatorial radius of the ellipsoids was around 31 Å and the polar radius between 13.2 and 16.8 Å. The modified geometry of β-aescin micelles with temperature may be the result of changed attractive or repulsive interactions, e.g., modified hydrogen bonds, with temperature. However, the clear cause for this behavior could not be identified within this work.

## 4. β-Aescin at Interfaces

### 4.1. Viscoelastic Properties at the Air-Water and Oil-Water Interface

Due to its amphiphilic character, β-aescin has a strong tendency to enrich at the air–water interface [37,38,41,44]. It was found that β-aescin adsorption layers feature an extremely high surface elastic modulus and very low gas permittivity which is important for the properties of foams and emulsions stabilized by this natural surfactant [40,41]. Golemanov et al. [37] studied the surface rheological properties of adsorption layers at the air–water interface. In their work, they investigate creep-recovery and oscillatory shear deformations of the adsorption layers. Among other triterpenoid and steroid saponins investigated, β-aescin was identified to have the highest elastic modulus together with other monodesmoside triterpenoid saponins. Generally, most of the triterpenoid saponins showed complex viscoelastic behavior with extremely high elastic modulus and viscosity. The origin of the viscoelastic properties of a β-aescin layer showing a domain structure, in which the domains are separated by mechanically weaker domain boundaries [45], was explained to be due to mainly 3 mechanisms: (I) sliding of β-aescin domains with respect to each other under shear stress, (II) viscoplastic deformation of the domain boundaries via migration of molecules along the domain sides, and (III) rearrangement of the molecules inside the domains for the restoration of their most favored intermolecular orientation, so that the energy of molecular interactions inside the domains is minimized. Additionally, they assume that the interactions between the saponin molecules are most probably governed by hydrogen bonds, and are possibly reinforced by hydrophobic interactions. From the relation between structure and surface rheological properties, the high surface elasticity of β-aescin was linked to its triterpenoid type oleanane aglycones. Such aglycones were discussed to promote denser packing and formation of strong inter-molecular bonds in the adsorption layers leading to high surface elasticity. The reported viscoelastic properties of β-aescin are 2400 mN/m for the surface shear elasticity and 130 Pa·s·m for the surface shear viscosity.

Pagureva et al. [41] elaborate on the viscoelastic properties of triterpenoid saponins and therewith of β-aescin. They investigate the relation between the molecular characteristics of the adsorption layers (from surface tension isotherms), and the surface rheological behavior (dilatational and shear deformation) of the same systems. In their study, they show for the group of monodesmoside and triterpenoid saponins (also β-aescin), that the saponin adsorption layers have high dilatational elasticity (≈100–160 mN/m), about 3 timers lower shear elasticity (≈30–55 mN/m), very high dilatational viscosity (≈80–120 mN s/m), and approximately 3-fold lower shear viscosity (from 28 to 35 mN s/m). These results denote, that β-aescin adsorption layers have distinct viscoelastic behavior. Therefore, saponin molecules like β-aescin are found to be able to form an adsorption layer which has a behavior of a viscoelastic material with a very high viscosity and elasticity. This property is lost for the crude horse chestnut extract [41]. The additional impurities and presence of other substances prevent the β-aescin molecules in this extract from dense packing.

**Figure 4:**
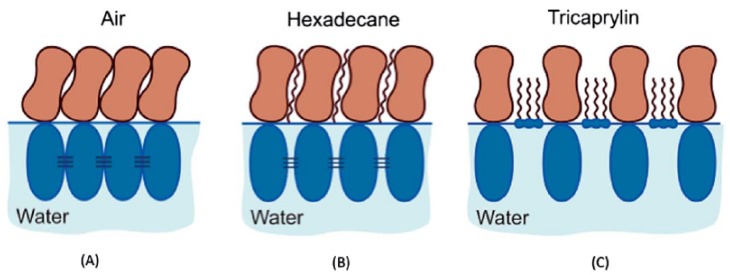
Proposed structure of saponin adsorption layers (**A**–**C**) for a monodesmosidic saponin such as β-aescin at (**A**) water–air and (**B**,**C**) water–oil (hexadecane and tricaprylin) interfaces. Reproduced from Reference [44] with permission from The Royal Society of Chemistry.

Later, Golemanov et al. [44] reported on the impact of a hydrophobic phase in contact with water on the interfacial properties of saponins, also of β-aescin. Generally, they show that the type of phase has a very strong effect on the interfacial elasticity and viscosity of the saponin adsorption layers and that it decreases in the order: air > hexadecane > tricaprylin. β-Aescin molecules are packed at the water–air interface in a side-on configuration (Figure 4A) with an area per molecule of around 0.5 nm${}^{2}$, leading to high viscoelasticities. However, when exposed to the water–oil interface not only the configuration but also the viscoelasticity decreases remarkably (Figure 4B,C). The modification of those was discussed to result from intercalated oil (hexadecane or tricaprylin) molecules in the adsorption layer which perturb strongly the packing of the monodesmosidic molecules at the interface, thus weakening the interactions between β-aescin molecules and disturbing the otherwise condensed packing of β-aescin molecules (Figure 4A–C). For instance, β-aescin showed comparable viscoelastic behavior at the water–air and water-hexadecane interface, whereas at the water-tricaprylin interface a non-elasticity monolayer with very low viscosity is obtained. This indicates a stronger disturbance of the intermolecular forces of the saponins aglycone by tricaprylin compared to hexadecane.

### 4.2. Gas Permeability in β-Aescin Adsorption Layers

**Figure 5:**
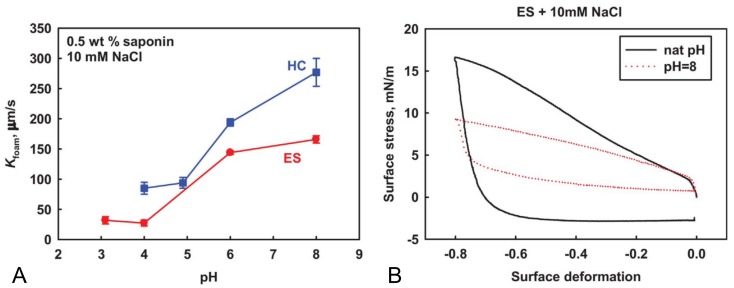
The figure shows in (**A**) the gas permeability ($K_{\mathrm{foam}}$) as a function of pH of the solution for foams stabilized by either the horse chestnut tree extract (HC, blue squares) and the pure saponin β-aescin (red dots). In (**B**) the surface stress is plotted against the surface deformation for β-aescin adsorption layers formed from solutions with natural pH (pH = 3.1) and adjusted pH of 8.0. Reprinted from Colloids and Surfaces A, 534, Tcholakova et al. [40], *Role of surface properties for the kinetics of bubble Ostwald ripening in saponin-stabilized foams*, pp. 16–25, ©2017, with permission from Elsevier.

Tcholakova et al. [40] studied the gas permeability and the Ostwald Ripening (OR) of β-aescin stabilized foams and foams stabilized by the horse chestnut (HC) extract. β-Aescin adsorption layers (at a low pH value of ≈3) exhibit a very large effective film thickness with very low gas permeability (≈30 μm/s) and a related very low rate of bubble Ostwald ripening in foams. Moreover, they report on a gas permeability for β-aescin foam films between single bubble and a large air–water interface of ≈100 μm/s. This value is an average over time since a noticeable decrease was observed from 250 μm/s down to 55 μm/s during the experiment. The authors suggest that this effect results from significant changes in the structure of adsorption layers. These structural changes may lead to lower surface tension of the bubble, decreased permeability of the adsorption layers, and/or other effects that reduce the rate of Ostwald ripening. The effective thickness of a liquid film for gas flux was determined from the experiments with a single bubble at the air–water interface. As a reminder, β-aescin exhibits very high dilatational and shear elasticity [37]. However, the horse chestnut (HC) extract containing only 20 wt% of saponin (from which 50% are β-aescin) according to the table in the supplementary information of this publication, showed a four-times higher gas permeability of the adsorption layers compared to pure β-aescin [40]. Tcholakova et al. identified several possible explanations for this: (I) the amount of β-aescin molecules in the mixtures differs significantly (95 % in the pure compound vs. 10 wt% in the HC mixture), (II) the different natural pH of the two saponin solutions studied (pH 4.9 for HC vs. pH 3.1 for β-aescin) leading to different structures of the adsorption layers, and (III) different concentrations of Ca${}^{2+}$ in the saponin extracts (0.17 mM in HC solution vs. 0.034 mM in β-aescin solution) being able to interact with the hydroxyl groups in the saponins. However, in order to clarify the importance of pH and Ca${}^{2+}$ concentration, the authors investigated the bubble Ostwald ripening as function of both parameters. The pH was varied between pH 3.1 and 8 and the results from the gas permeability ($K_{\mathrm{foam}}$) as a function of pH of the solution are shown in Figure 5A.

The results clearly indicate a pH dependence of the gas permeability in foams stabilized by β-aescin and HC, showing qualitatively the same dependence for both systems. However, the lower $K_{\mathrm{foam}}$ for β-aescin stabilized foams indicates strong dependence on the structure of the adsorption layers, most probably caused by partial ionization of the carboxylic group in the β-aescin molecules at the higher pH values (see $pK_{a}$ value of 4.7 ± 0.2 as determined for β-aescin by Dargel et al. [29]). The electrostatic repulsion between the adsorbed saponin molecules would hinder the formation of densely packed adsorption layers (see Figure 5B). The increase of pH up to 8 leads to a loose packing of the respective adsorption layers and therewith an increase of the corresponding gas permeability up to a value of ≈150 μm/s. The authors found, contrarily to the observations as a function of pH, that the Ca${}^{2+}$ concentration (from concentration variation experiments) only has a minor effect on the rate of Ostwald Ripening and interfacial properties of the β-aescin and HC adsorption layers. Nevertheless, the similar behavior and shape of the curves of both extracts confirms that β-aescin is the main surface-active compound in the HC mixture which is modified by the Ca${}^{2+}$ concentration. Therefore, the most important parameters leading to the different properties of β-aescin and HC adsorption layers are the different pH values of the solutions used and the admixtures in the HC extract distorting the dense packing of molecules at the water–air interface.

### 4.3. Structure of the β-Aescin Monolayer at the Air-Water Interface

The structure of the adsorbed layers at the water–air interface was determined by Penfold and coworkers using a combination of neutron reflectometry (NR) and surface tension measurements [38]. The investigations were performed from below to above the cmc of β-aescin and at different pH values, as the pH significantly impacts the constitution of the adsorbed monolayers [40]. From the determination of the surface coverage, the authors identified the area per β-aescin molecule to 0.75, 0.69, and 0.61 ± 0.04 nm${}^{2}$ at pH 8, *natural* pH, and pH 4, respectively. Furthermore, the area/molecule at a concentration above the cmc is independent of pH and has a mean value of ≈0.69 ± 0.04 nm${}^{2}$. These results indicate that the behavior of β-aescin differs from the one of a simple nonionic surfactant. The carboxylic group in the glycone of β-aescin (see Figure 2) is expected to be protonated at pH 4, ionized at pH 8, and probably partially ionized at natural pH.

**Figure 6:**
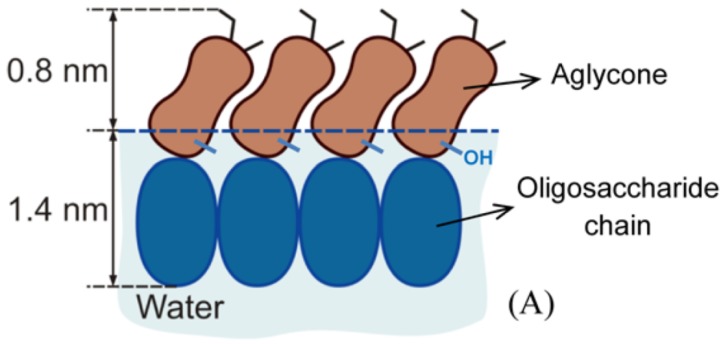
Schematic of the proposed orientation of densely packed β-aescin molecules at the water–air interface, as determined from NR experiments. Reprinted with permission from Penfold et al. [38], ©2018 American Chemical Society.

A more detailed characterization of the adsorbed monolayer was done by NR experiments where different solvent contrasts and a simultaneous analysis of the recorded data were applied in order to give good results of the structure of the adsorbed monolayer. The best description of the NR data was achieved when describing the adsorbed layer by a two-layer model. A schematic showing the orientation of the β-aescin molecules at the air–water interface is depicted in Figure 6.

It was found that the outer layer adjacent to the air phase corresponds to the triterpenoid hydrophobic region of the saponin molecules and has a thickness of ≈0.8 nm (and a volume fraction of saponin of ≈0.3 containing no solvent). However, the total length of this hydrophobic region is ≈1.4 nm. Hence, either a fraction of the hydrophobic scaffold must be embedded in the aqueous subphase and/or the triterpenoid scaffold is strongly tilted with respect to the water–air interface. Both explanations are consistent with a recent simulation of β-aescin adsorption by molecular dynamics [42]. The inner layer, adjacent to the aqueous phase, was found to have a thickness of ≈1.4 nm. This layer contains both water molecules and saponin subunits, with volume fractions of ≈0.2 and ≈0.8, respectively. However, the high volume fraction of β-aescin parts in this inner layer (80 vol%) reveals that it is composed mostly of tightly packed, hydrated sugar groups. Therefore, this study confirmed that the saturated adsorption layers are governed by densely packed hydrophilic saccharide groups, i.e., the head groups of β-aescin. This was already discussed in other works [37,40,41] and is now confirmed and quantified in this study. The tight molecular packing and the strong hydrogen bonds between the neighboring saccharide groups are probably the main reasons for the unusual rheological properties of the saponin adsorption layers.

### 4.4. MD Simulations of a β-Aescin Monolayer

**Figure 7:**
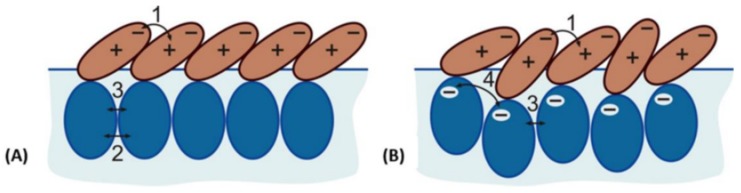
Proposed arrangement of β-aescin molecules of (**A**) neutral and (**B**) ionized molecules in a surface cluster adsorbed at a water-vacuum interface, as obtained from MD simulations. The numbers 1–4 indicate the main inter-molecular forces dominating the β-aescin self-assembly. These are: (1) long-range attraction due to the inhomogeneous charge distribution in the aglycone and short-range dispersion (London) van der Waals forces between the aglycone fragments; (2) intermediate-range dipole-dipole/H-bond interaction, (3) short-range classical H-bonds, and (4) water-screened electrostatic repulsion between the charged carboxyl groups in the ionized form of β-aescin molecules. Reprinted with permission from Tsibranska et al. [42], ©2017 American Chemical Society.

Tsibranska et al. [42] performed classical atomistic dynamics simulations of neutral and ionized β-aescin molecules adsorbed at the vacuum–water interface (water with 10 mM NaCl) in order to clarify their orientation and interactions on the water surface. Thereby, the neutral form corresponds to β-aescin molecules at e.g., pH 4 and the ionized form to β-aescin at e.g., pH 8 when compared to experimental findings. Significant differences in the behavior of the neutral and the charged β-aescin molecules are observed. It was e.g., found that the neutral form rapidly assembles in a compact and stable surface cluster whereas the ionized form assembles in a much slower manner. The rapid assembly of the neutral form was explained by the action of long-range attraction between the hydrophobic aglycones, combined with intermediate dipole-dipole attraction and short-range hydrogen bonds between the sugar residues in β-aescin molecules. The long-range attractive force leading to cluster formation is thought to originate from the aglycone part not immersed in water (see Figure 6 and Figure 7). It also orients the β-aescin molecules with respect to each other.

Additionally, a charge distribution was identified over the β-aescin molecule in the aglycones of both forms. This finding implies the appearance of electrostatic attraction between the oppositely charged molecular fragments in the neighboring surfactant molecules when they are properly oriented with respect to each other. This attraction is a kind of multipole-multipole intermolecular interaction and is probably causing the fast formation of the molecular clusters at the interface. Moreover, it implies the molecules to assemble in a preferred mutual orientation that maximizes this intermolecular attraction. When aglycones are close, also interactions via dispersion (London)-type attractive forces (van der Waals attraction) between large molecular fragments occur. However, in the ionized form, the deprotonated carboxylic group causes the presence of a large negative charge on the hydrophilic part immersed in water. At the same time, the aglycone appears the same for both forms of β-aescin so that also for the ionized form the long-range van der Waals attraction between the aglycones is expected. However, this interaction would be superimposed by a long-range electrostatic repulsion of charged glyconic fragments which explains the slower aggregation of ionized β-aescin molecules and the lower stability of the largest clusters formed by the ionized molecules. Furthermore, in the ionized form more water molecules are located around the charged carboxylic acid residue reducing the overall attractive strength between β-aescin molecules.

The structure of the adsorption layers slightly differs for the uncharged and charged β-aescin molecule, again emphasizing the importance of controlled pH during experiments. The *maximum* thickness of β-aescin molecules (and *most probable* thicknesses) reported in the normal direction is 2.24 nm (0.88 nm) for single neutral β-aescin, 2.68 nm (1.05 nm) for a single ionized β-aescin, 2.75 nm (1.08 nm) for the neutral β-aescin cluster, and 3.24 nm (1.27 nm) for the ionized β-aescin cluster. It is observed that the anions are thicker compared to the neutral form. Moreover, the molecular length is 2.22 nm for the neutral and 2.11 nm for the charged one. More interesting is that the length of the β-aescin molecules is longer than the projected thickness normal to the interface which only may happen if the molecules are tilted with respect to the interface. Such a tilt was found for the aglycone in the neutral form by 110 ± 15 ${}^{\circ }$ and in the ionized form by 116 ± 15 ${}^{\circ }$. The hydrophilic glyconic part is located at around 160 ${}^{\circ }$ with respect to the aglycone, which is valid for the neutral and ionized form of β-aescin. In summary, the glyconic part is much more mobile and flexible undergoing spontaneous conformation changes. Nevertheless, short-range classical H-bonds between sugar residues lock the orientation of β-aescin molecules. The possible orientations and interactions in the neutral and ionized form are summarized in Figure 7A,B, respectively. Whereas in the neutral form, three types of attractive interactions are present, in the ionized form the long-range attraction is counteracted by the electrostatic repulsion between the charged carboxylic groups in the sugar residues. These interactions are also expected to control the viscoelastic properties of β-aescin adsorption layers and therefore, also the differences observed between neutral and ionized form of β-aescin.

The work on the adsorption from dilute solutions of β-aescin was very recently complemented by works on much more dense layers by the same group [43]. Compared to the rather dilute system, the concentrated layers show slight differences. β-Aescin molecules are less submerged in the water phase and adopt a more upright position compared to the dilute layers. This occurs in order to reduce the intermolecular steric repulsion and to attain tight packing of the aglycones and optimum inter-surfactant hydrogen bonding. Consecutively, the adsorption layers were found to deform and become heavily curved. Additionally, trapped water molecules are located around the glycones placed above the water surface to solvate them in the dense layer. However, the simulations revealed an attained long-range order and a collective behavior of all β-aescin molecules on the surface without achieving the state of a true crystal structure.

## 5. β-Aescin Interaction with Bare Lipid Model Membranes

The interaction of β-aescin with bare lipid model membranes from the physicochemical perspective is subject of works published over the last 2 years by Sreij et al. [46,47,48] and Geisler et al. [49]. The work is done exemplarily on phospholipid bilayers composed of 1,2-dimyristoyl-*sn*-glycero-3-phosphocholine (DMPC) at constant environmental conditions, i.e., in 50 mM phosphate buffer at physiological p(H)≈ p(D) of about 7.4 and a DMPC mass concentration of *w*(DMPC) = 15 mg/mL. All samples were prepared by the lipid film hydration method; i.e., the dry thin lipid film is hydrated by a β-aescin containing buffer solution and subsequently exposed to five freeze-thaw cycles. The β-aescin concentrations investigated range from 0 (pure lipid bilayers) to 30 mol% β-aescin molecules with respect to the number of DMPC molecules. Here, β-aescin concentrations below and above cmc (β-aescin) are covered. The cmc is crossed at about 3–4 mol% of β-aescin with respect to the lipid. The interaction of the lower contents was generally studied on bilayers in the form of extruded small unilamellar vesicles (SUVs). Polycarbonate membranes for extrusion had a pore size of 500 Å.

The successful incorporation of β-aescin into the bilayer of the samples prepared as mentioned above was shown by a changing cross-sectional electron density profile of samples with varying β-aescin concentration obtained from SAXS measurements [47].

**Figure 8:**
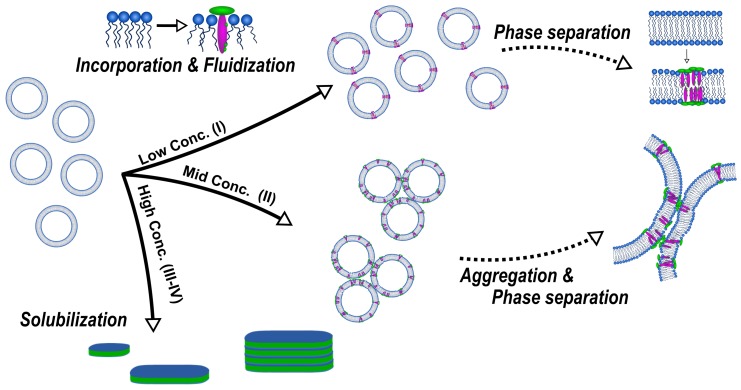
Summary of β-aescin-DMPC interactions depending on saponin concentration. The incorporation of β-aescin molecules into the DMPC bilayer increases the bilayer fluidity and β-aescin molecules phase-segregate into saponin-rich and saponin-poor domains in the membrane. With increasing β-aescin concentration, vesicles start to become unstable in solution and agglomerate due to the formation of larger domains. At β-aescin concentrations far above cmc of β-aescin (high conc. regime (III–IV)) the DMPC membrane is solubilized by the saponin molecules and discoidal bicelles form which are tunable in size by composition and temperature. Adapted with permission from Sreij et al. [46], ©2017 American Chemical Society.

In these works, these authors elucidated a concentration- and lipid phase state-dependent structure formation caused by the interaction of β-aescin with the DMPC bilayer. In general, they divided the studied range into three parts (III and IV belong to the same concentration range), similar to the three-stage model of membrane solubilization by classical detergents and bile salts [50,51,52], whereby each of them represents a concentration regime where similar interactions and structures are obtained. An overall summary of the concentration-dependent interactions and structures formed is illustrated schematically in Figure 8.

At low β-aescin contents (region I) which ranges up to ≈0.8 mol%, β-aescin can be incorporated into stable SUVs and the structural parameters of these SUVs were studied by small-angle scattering with X-rays (SAXS) and neutrons (SANS) [47]. It was found that β-aescin incorporation leads to higher radii of gyration ($R_{G}$) with rising β-aescin content while the vesicles temperature-driven phase transition due to the conformational transition of DMPC’s alkyl chains remains conserved. The membrane thickness is only slightly affected by β-aescin incorporation.

**Figure 9:**
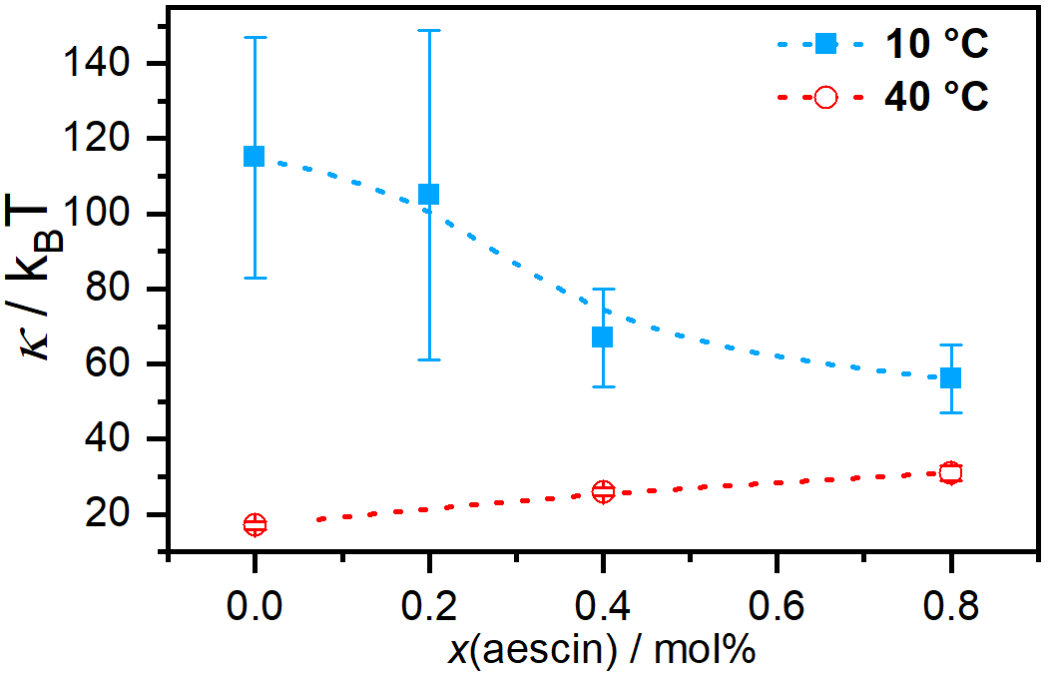
Bilayer bending modulus κ as function of β-aescin content for 10 ${}^{\circ }$C and 40 ${}^{\circ }$C. Dotted lines are guides to the eye. Reproduced from Ref. [47] with permission from the PCCP Owner Societies.

Membrane dynamics affected by the incorporation of β-aescin were addressed by Sreij et al. [47] by means of neutron spin-echo spectroscopy (NSE) measurements for DMPC SUVs in the mentioned β-aescin content regime. For β-aescin, the bilayer bending modulus κ depends on β-aescin concentration and lipid phase state. For the phospholipid DMPC, the main phase transition occurs at a temperature of $T_{m}=$23.6 ${}^{\circ }$C [46]. In the gel phase of DMPC, i.e., at 10 ${}^{\circ }$C, the interactions of β-aescin with the bilayer lower the bending modulus and the membrane softens in a concentration-dependent manner (Figure 9).

Above the melting temperature of DMPC, the bilayer rigidifies in presence of β-aescin and the value of κ increases. To explain these effects, the authors used analogies to similar molecules since there are no comparable experiments on saponin molecules. The softening most likely results from headgroup-headgroup interactions between the saponins sugar moieties and DMPCs phosphocholine groups, similar to the effect of nonsteroidal anti-inflammatory drugs (NSAIDs) known to interact with the headgroups of phospholipid bilayers [53]. This becomes especially convincing when taking into account that both, the NSAID molecules and also the β-aescin used in this work, are deprotonated and carry a single charge. The stiffening effect at the phospholipids fluid phase was explained by the insertion of the stiff triterpene backbone of β-aescin into the flexible bilayer, similar to the known effect of cholesterol incorporation.

**Figure 10:**
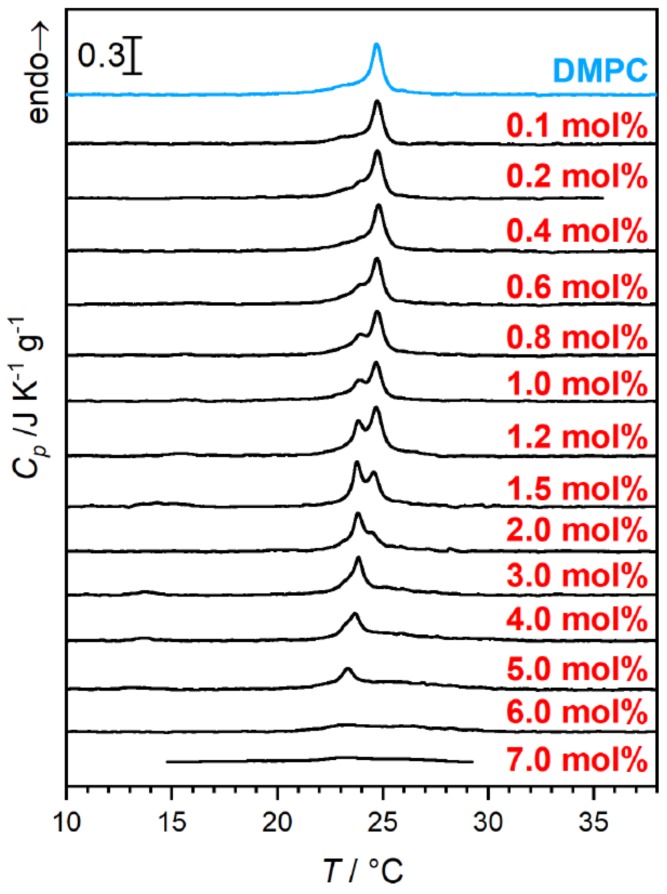
DSC of β-aescin containing samples. The red numbers denote β-aescin concentration in mol% with respect to the molar amount of used DMPC. An increasing β-aescin concentration enhances phase segregation and shows in the formation of a second peak at lower temperature. The experimental procedure is given elsewhere [21,48,55]. Adapted from Ref. [48] with permission of Elsevier.

Upon rising the β-aescin content to values higher than ≈1 mol%, β-aescin incorporation increases the effect of phase separation inside the lipid bilayer. This shows clearly in the endotherms measured by differential scanning calorimetry (DSC) (Figure 10) and was moreover confirmed by wide-angle X-ray scattering (WAXS) experiments [46]. The formation of saponin-poor and saponin-rich domains becomes obvious in the presented data and is expressed by the rising intensity of the peak at lower temperature. Similar effects resulting from headgroup-drug interactions were observed for non-steroidal anti-inflammatory drugs (NSAIDs) known to interact with phospholipids headgroups from the inside of the bilayer [54]. The presence of the stiff saponin backbone in the bilayer distorts the temperature-driven cooperative phase transition of DMPC molecules and the bilayer becomes fluidized at lower temperatures [21]. At around 6 mol% of the saponin the signal broadens significantly and is only weakly visible indicating different structures in the samples.

**Figure 11:**
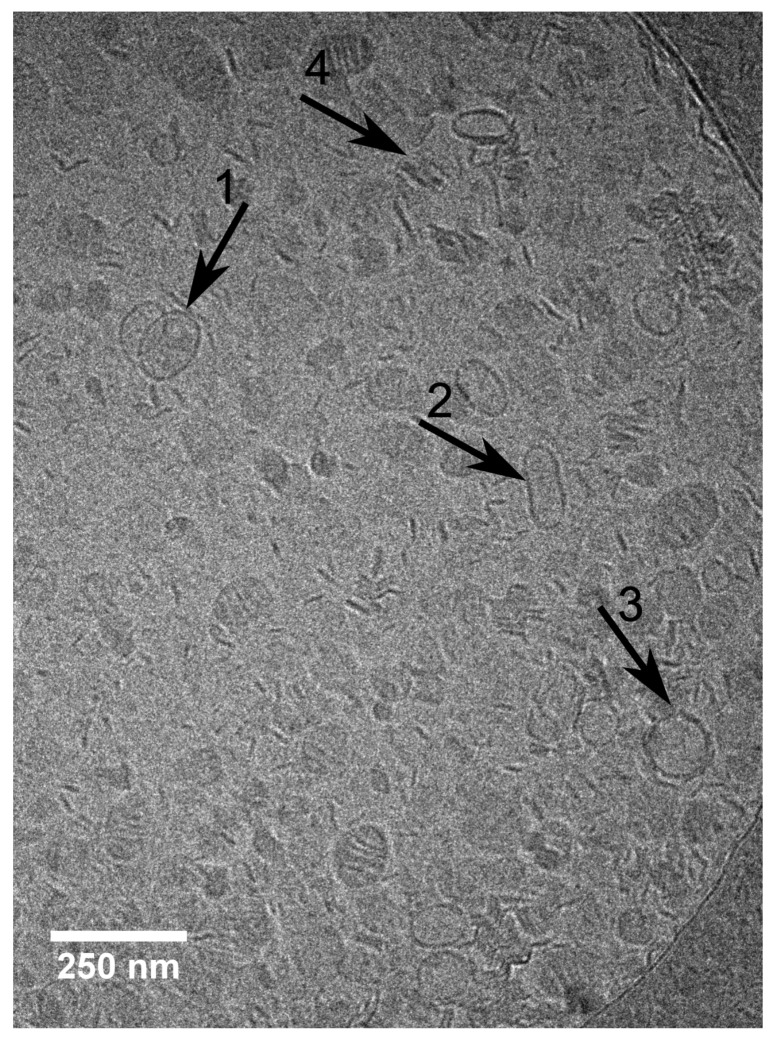
Cryo-TEM image of a sample with 4 mol% β-aescin prepared at 10 ${}^{\circ }$C. The arrows point on deformed SUVs (1), elongated SUVs (2), SUVs with edges (3) and stacks of bilayer disks (4). Reproduced from Ref. [48] with permission of Elsevier.

A study devoted to the “mid” concentration regime between 1 and 7 mol% [48] (region II) showed that an increasing β-aescin concentration leads to aggregated SUVs which start to deform and solubilize at concentrations around cmc (β-aescin). The diverse structures simultaneously present in this regime are visualized by cryogenic transmission electron microscopy (cryo-TEM) for a sample with 4 mol% β-aescin (Figure 11). The figure shows the manifold of coexisting structures in the “mid” concentration regime, as this concentration is at the onset of complete decomposition into free-standing discoidal micelles, also called “bicelles” or “nanodisks”. At this β-aescin concentration, the presence of β-aescin leads to deformation (arrow 1), elongation (arrow 2) of and the presence of peculiar features like edges (arrow 3) in gel phase DMPC SUVs. Additionally to this, also a certain fraction of already solubilized small membrane fragments/bicelles is visible (arrow 4).

**Figure 12:**
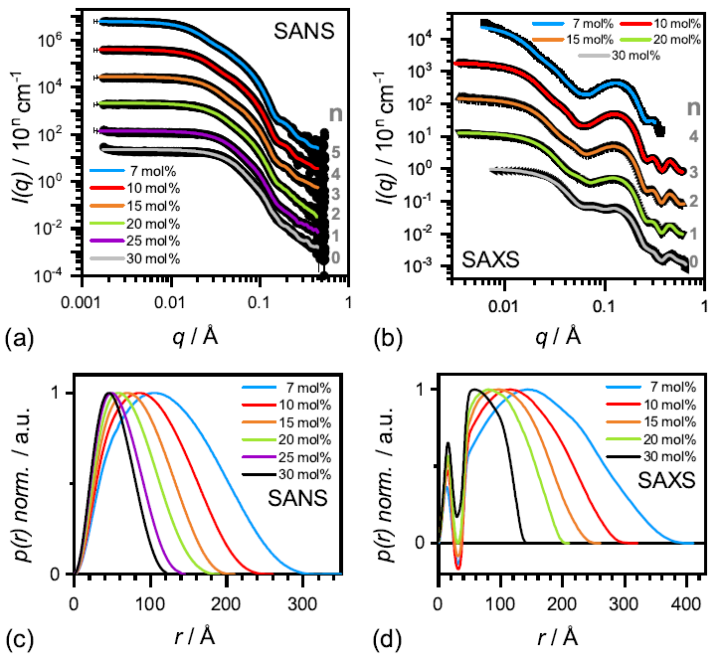
SANS (**a**) and SAXS (**b**) curves of β-aescin stabilized bicelles at 10 ${}^{\circ }$C for different aescin concentrations. The solid lines are fits obtained by using the IFT method developed by O. Glatter. (**c**) Normalized spatial correlation functions p(r) from SANS and (**d**) from SAXS from the respective panels (**a**) and (**b**). The legend indicates the β-aescin content in mol% with respect to the amount of DMPC. Reproduced with permission from Geisler et al. [49], ©2019 American Chemical Society.

The effect of complete solubilization of a DMPC membrane at β-aescin contents >7 mol% (region III), was subject of a recent work published by Geisler et al. [49]. This work has shown that well-defined bicelles form at β-aescin concentrations far above its cmc and at a temperature below the lipids $T_{m}$. Thereby, the disk radius is determined by the number of β-aescin molecules and decreases with increasing β-aescin content from around 350 Å at 7 mol% to 120 Å at 30 mol% β-aescin (Figure 12). Disk radii were obtained from SAXS and SANS. The bicelles result to be tunable in size by composition. Moreover, the authors identified the onset of a fourth (IV) regime at around 25 mol%, where the radius of the bicelles becomes smaller than the thickness of the bilayer. Above this β-aescin content, a slightly different interaction with the bilayers is expected.

**Figure 13:**
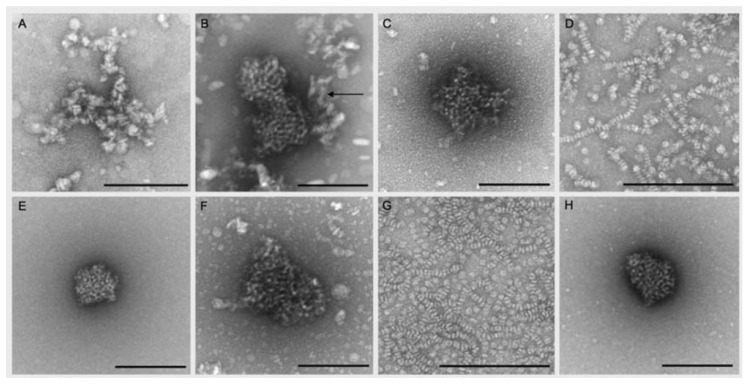
TEM images of diverse samples. (**A**) β-aescin micelles in water. (**B**–**H**): Pseudo-ternary and pseudo-binary systems prepared by the lipid film hydration method with aqueous solution. (**B**,**C**) DPPC:β-aescin:cholesterol mixture with a mass ratio of 1:3:1 (**C**) storage period of 2 months). (**D**) DPPC:β-aescin mixture with a mass ratio of 1:3. (**E**) cholesterol:β-aescin mixture with a mass ratio of 1:3. (**F**,**G**) TEM images of pseudo-ternary and pseudo-binary systems prepared by lipid film hydration method and hydration with Tris buffer (pH 7.4, 140 mM). (**F**) DPPC:β-aescin:cholesterol mixture with a mass ratio of 1:3: 1. G) DPPC:β-aescin mixture with a mass ratio of 1:3. (**H**) Cholesterol:β-aescin mixture with a mass ratio of 1:3. The scale bar equals to 200 nm. Reproduced from Reference [39] with permission of Planta Medica, © Georg Thieme Verlag KG.

It was found that the β-aescin disks self-assemble at low temperature, i.e., at temperatures where the phospholipid is in its rigid gel state. At this lipid phase state and temperature (10 ${}^{\circ }$C), the phospholipid chains of DMPC are tightly packed and the formation of intermolecular hydrogen bonds (involving lipid and β-aescin molecules) is especially favorable. Here, the model assumes that the bicelles have circular discoidal shape with a surrounding rim composed of β-aescin molecules. The size of the discoidal structures is determined by the amount of β-aescin molecules. At a lower β-aescin-to-DMPC ratio, larger structures are stabilized and vice versa. Moreover, there are indications that the bicelles undergo a temperature-dependent phase transition into large vesicles and stacked bilayers [48,49], similar to phospholipid-bile salt systems [56]. In addition to the DMPC-β-aescin system, solubilization of a 1,2-dipalmitoyl-*sn*-glycero-3-phosphocholine (DPPC) membrane by β-aescin in the lipids gel-phase was reported in a study of de Groot et al. [39]. By TEM, small and oval shaped structures, partially stacked in a worm-like arrangement were found for a DPPC-β-aescin mixture in a ratio of 1:3. These structures are depicted in Figure 13D,G and also correspond to the formation of bicelles.

## 6. Synergies of β-Aescin, Cholesterol and Drug Molecules

The effect of cholesterol addition on the structural parameters of β-aescin-containing phospholipid bilayers composed of DMPC was investigated by Sreij et al. [55]. In this work, the cholesterol contents studied were 1, 5, and 10 mol% and the β-aescin contents ranged from 0 to 6 mol% with respect to the amount of DMPC in solution. Thereby, the formation of β-aescin-cholesterol complexes in DMPC bilayers is confirmed which was already shown for other saponins. The complex formation was investigated by various methods, including SAXS & SANS, wide-angle X-ray scattering (WAXS) and differential scanning calorimetry (DSC). DSC confirmed complexation of cholesterol with β-aescin. This was revealed by a modification of the endothermic signal whereas the effect increases with rising steroid content. Although the lowest amount (1 mol% of cholesterol) seems to reduce the β-aescin-driven phase segregation, 5 and 10 mol% cholesterol lead to strong broadening of the endothermic peaks, resulting from the distortion of the cooperative phase transition of hydrocarbon chains through insertion of the stiff steroid molecules. WAXS data show that already 0.5 to 1 mol% of β-aescin are able to reverse the distorting effect of 10 mol% cholesterol on the WAXS pattern. Moreover, it could be shown that $T_{m}$ is generally lowered by about 1 ${}^{\circ }$C and the area per headgroup ($A_{L}$) increased by around 0.3 Å${}^{2}$ upon cholesterol (10 mol%) addition. SAXS and SANS demonstrate how β-aescin–cholesterol complexes modify structural parameters of SUVs investigated. It becomes visible that the structural parameters (e.g., inner core radius and bilayer thickness) are dominated by the effects resulting from β-aescin. Nevertheless, the presence of cholesterol enhances inter-vesicular effects such as aggregation of SUVs.

De Groot et al. [39] investigated the interaction of β-aescin with DPPC and cholesterol at relatively high lipid-to-cholesterol-to-saponin ratios inspired by the quantities reported for the formation of immune stimulating complex matrices (ISCOMs). In their work, the authors report on the formation of circularly and ovally shaped structures in either water or Tris buffer solution at pH 7.4. TEM images illustrating these structures (Figure 13) reveal that the final form of aggregates obtained strongly depends on composition. β-Aescin micelles (in water) exhibit circular shape (A) and the binary DMPC/β-aescin mixtures (panels D & G) form discoidal structures which assemble into worm-like aggregates. This finding is in agreement with the results on bicelle formation in DMPC-β-aescin mixtures reported by Geisler et al. [49]. The ternary system (panels B & C), on the other hand, leads to the formation of slightly different structures. These are significantly larger and posses partially oval shape (arrow in panel B). The authors suggest that these structures are involved in the ISCOM formation process. However, another interesting observation is that similar but almost spherical aggregates are obtained in the binary β-aescin-cholesterol mixtures (panels E & H) in the absence of DPPC, indicating that cholesterol is completely solubilized by β-aescin. The comparison of the TEM images also suggests that DPPC has a major influence on the alignment of the flat disks in solution.

The reverse effect to the one of cholesterol was found for the non-steroidal anti-inflammatory drug (NSAID) ibuprofen [21] at similar experimental conditions. In this work, which was conducted at pH 7.4 in a 50 mM phosphate buffer solution, both, the β-aescin and ibuprofen molecules are deprotonated. The β-aescin-contents range from 0 to 6 mol% and ibuprofen was set constant at either 5 or 10 mol%, all with respect to the amount of DMPC. Contrarily to the effect of cholesterol, NSAIDs in general and therefore also ibuprofen, are known to interact with the phospholipid headgroups from the inside of the bilayer. The study was again conducted by means of SAXS, WAXS, and DSC. In contrast to the behavior with cholesterol, strong complexation of β-aescin and the ibuprofen molecules was not found. Nevertheless, synergistic effects of both types of molecules were observed on the structural parameters of DMPC bilayer. DSC showed that the concentration-dependent phase segregation of β-aescin-molecules in the bilayer is affected by the presence of ibuprofen. A general shift of the entire endothermic signal to generally lower temperatures is observed. This effect enhances with rising ibuprofen content. In addition to this, the peak at lower temperatures caused by β-aescin (Figure 10) forms first at even lower β-aescin contents compared to the NSAID-free system and is still observed for the highest saponin-contents studied, compared to the NSAID-free system. WAXS, which investigates the acyl chain correlations and gives therefore information about the effect on the lateral arrangement of DMPC molecules, shows that the effect of ibuprofen (10 mol%) is different from that of cholesterol. Here, contrarily to the steroid effect, no prominent impact on area per lipid molecule $A_{L}$ could be shown. $A_{L}$ is obtained from WAXS as described by Equation (3) in Reference [21]. Therefore, no direct complexation of these two molecules is seen. However, it could be shown that the presence of ibuprofen decreases $T_{m}$ especially at lower β-aescin (<1 mol%) contents. This effect becomes negligible at 5 mol% of the saponin. SAXS experiments revealed that the β-aescin concentration dominates the vesicle size parameters obtained. In addition, the most prominent effect is observed in a temperature-dependent study conducted from 10 to 50 ${}^{\circ }$C at 5 mol% β-aescin and 10 mol% of the NSAID. Here, and contrarily to the aggregation effect of cholesterol, it could be shown that ibuprofen reduces the amount of aggregation between SUVs and bilayers in general. More precisely, high amounts of ibuprofen could reduce the temperature-driven membrane stacking as observed for the binary β-aescin-DMPC mixtures, especially for the higher temperatures. Already 10 mol% ibuprofen leads to the formation of free-standing and extended membrane sheets whose size exceeded instrument resolution.

## 7. Interaction of β-Aescin with Langmuir Monolayers from Langmuir Tensiometry (LT) Experiments

Up to now, there are only a few reports on the effect of β-aescin on a DMPC monolayer [39,46]. Sreij et al. [46] studied the penetration effect of β-aescin into a DMPC monolayer by subphase exchange experiments. Thereby, the DMPC monolayer was adsorbed at the buffer–air interface and was studied in either the liquid condensed (LC) or liquid expanded (LE) phase at 4 ${}^{\circ }$C or 38 ${}^{\circ }$C, respectively. In these subphase-exchange experiments β-aescin gently incorporates into the adsorption layer from the bulk solution.

**Figure 14:**
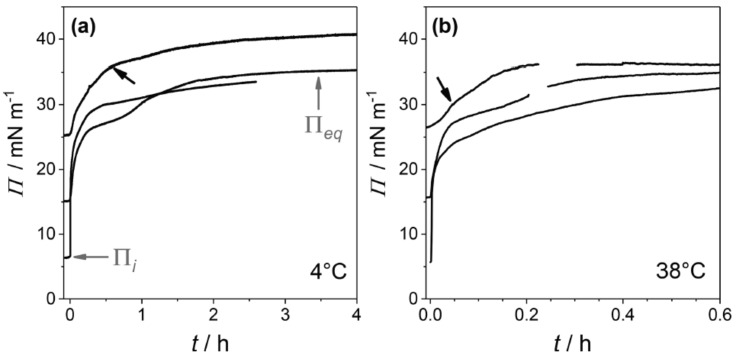
Surface pressure-time adsorption kinetics for a DMPC monolayer after the start of buffer sub-phase exchange (50 mM phosphate buffer, pH 7.4) against β-aescin solution (same conditions) until reaching equilibrium pressure $\Pi_{eq}$. The experiments were carried out at (**a**) 4 ${}^{\circ }$C and (**b**) 38 ${}^{\circ }$C for different initial pressures $\Pi_{i}\approx$ 5, 15, and 25 mNm${}^{-1}$. The arrows mark an intermediate cusp which is explained in the main text. Reproduced with permission from Sreij et al. [46], ©2017 American Chemical Society.

**Figure 15:**
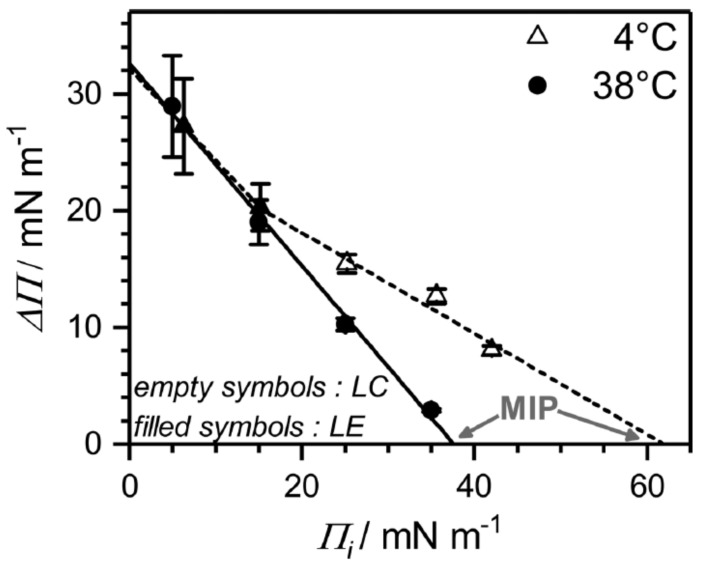
Results related to the surface pressure-time adsorption kinetics shown in Figure 14. The figure shows the insertion pressure ($\Delta \Pi =\Pi_{eq}-\Pi_{i}$) upon β-aescin incorporation into a DMPC monolayer as a function of initial pressure $\Pi_{i}$ obtained from Π(t) adsorption kinetics. The temperatures investigated are 4 ${}^{\circ }$C (triangles) and 38 ${}^{\circ }$C (dots). Filled symbols: LE phase; open symbols: LC phase. MIP (grey) denotes the maximum insertion pressure (MIP) at ΔΠ=0. Reproduced with permission from Sreij et al. [46], ©2017 American Chemical Society.

From surface pressure-time (Π−t) adsorption kinetics faster adsorption at higher temperature, which is due to faster diffusional adsorption of β-aescin from the solution, was found (Figure 14). In these Π−t-kinetics a special feature (an intermediate cusp) was found after initial rapid pressure increase and before reaching the equilibrium surface pressure $\Pi_{eq}$. Based on this finding, the authors assume that an adsorption barrier arises leading to a transient meta-stable state before reaching the final penetration/reorganization of β-aescin inside the DMPC monolayer, or transient domain-formation within the monolayer, or both with time. This effect was found to take longer time at low than at high temperature (Figure 14). Moreover, it was found that penetration of β-aescin into a disordered LE phase (e.g., at weak initial compression of the lipid film) is facilitated and independent of temperature (Figure 15). This shows in the pressure difference $\Delta \Pi =\Pi_{eq}-\Pi_{i}$ which is calculated as a free energy difference between the initial pure lipid state ($\Pi_{i}$) and the final equilibrium plateau ($\Pi_{eq}$) corresponding to the mixed β-aescin-lipid monolayer after β-aescin has penetrated the DMPC monolayer from the solution. From the maximum insertion pressure (MIP) it becomes evident, that the strength of penetration depends on the phospholipid phase state. The LC phase is much more penetrable (MIP${}_{\mathrm{LC}}\approx$62 ± 2 mNm${}^{-1}$) by β-aescin than the LE phase (MIP${}_{\mathrm{LE}}\approx$38 ± 3 mNm${}^{-1}$). From these results the authors conclude that the higher MIP value may be the result of a more pronounced impact of β-aescin molecules in the gel-phase bilayer-like LC phase than in the LE phase. However, an alternative explanation which was suggested by one of the referees of the present review differs from this explanation. He pointed out that a different explanation might be concluded from the higher slope of the surface pressure isotherm in the LC state than in the LE state. Due to the steepness of the curve even small increases of surface concentration (decrease of area per molecule) could result in a huge jump of surface pressure.

De Groot et al. [39] studied the interaction of β-aescin with DPPC and cholesterol monolayers and combinations of both also by Langmuir tensiometry (LT). In their experiment they compare the adsorption of β-aescin, which is injected in the subphase, with either a DPPC or a cholesterol monolayer or a mixture of both. The work was carried out at 20 ${}^{\circ }$C and the subphase-exchange experiments were recorded for a duration of 1000 s, i.e., 16.6 min. They found that the most prominent effect of β-aescin in the subphase occurs on cholesterol-containing monolayers confirming the strong affinity of β-aescin and cholesterol molecules. These results are in concordance with the effects of β-aescin on DMPC bilayers in the presence of cholesterol as discussed previously [55] (Section 6).

## 8. Conclusions

The results summarized in this review related to the self-assembly of β-aescin in solution as well as at interfaces and the interaction with lipid-model membranes show special interactions caused by the specific molecular structure of the saponin β-aescin. In particular, several studies show the influence of the pH and thus the protonation state of β-aescin on the self-assembly of β-aescin. A deprotonation of the acidic residue in the glyconic molecular part drastically modifies the properties of β-aescin monolayers at interfaces and in foams. This knowledge plays an important role in the various applications of β-aescin, e.g., in the cosmetics sector.

The interaction of β-aescin with phospholipid model membranes further demonstrated decomposition and solubilisation into small lipid membrane fragments. The formation of bicelles at high β-aescin concentration may have potential in the use of bicelles for the structural determination of membrane proteins by nuclear magnetic resonance (NMR). Furthermore, results from studies with membrane additives such as cholesterol and ibuprofen can provide important, fundamental insights that help to better understand the impact of β-aescin on the human body. This is particularly important as β-aescin is present in a variety of freely available and advertised products, but the background is unknown for many of the observed beneficial effects. In this context, the results presented in this review can serve as a basis for model systems closer to the human organism. This includes, for example, the elucidation of the interactions of β-aescin with animal tissues such as skin.

Moreover, the reviewed works reveal that β-aescin shows strong synergistic effects with a drug like ibuprofen. This effect should be further elucidated and it would be of great interest to relate this found molecular interaction to changes in efficiency of the drug. Also, other NSAIDs should be evaluated in this context. Exploiting such synergies could lead to reduced doses of drugs with e.g., less burden for the liver.

## Figures and Tables

**Table 1:** Critical micelle concentrations (cmc) of β-aescin. (Used abbreviations: PB: phosphate buffer, T: tensiometry, AF: autofluorescence.)

Reference	T/${}^{\circ }$C	cmc	Solvent	pH	Method
Pekdemir et al. [35] (1999)	30	0.78 mM	water	9.0	T
Pekdemir et al. [35] (1999)	30	1.69 mM	3 M urea	9.0	T
Pekdemir et al. [35] (1999)	30	2.20 mM	3 M glucose	9.0	T
Pekdemir et al. [35] (1999)	30	0.47 mM	sea water	9.0	T
Böttger et al. [36] (2012)	20	0.088 mM	0.1 M PBS	-	T
Golemanov et al. [37] (2013)	-	0.008 wt%	H${}_{2}$O	-	T
Penfold et al. [38] (2018)	20	0.06 mM	H${}_{2}$O	4	T
Penfold et al. [38] (2018)	20	0.11 mM	H${}_{2}$O	*natural*	T
Penfold et al. [38] (2018)	20	0.11 mM	H${}_{2}$O/D${}_{2}$O (92/8 mol%)	*natural*	T
Penfold et al. [38] (2018)	20	0.072 mM	H${}_{2}$O	8	T
De Groot et al. [39] (2018)	25	0.065 g/L (≈0.057 mM)	H${}_{2}$O	-	T
Dargel et al. [29] (2019)	10	0.38 ± 0.09 mM	50 mM PB	7.4	AF
Dargel et al. [29] (2019)	15	0.38 ± 0.10 mM	50 mM PB	7.4	AF
Dargel et al. [29] (2019)	20	0.38 ± 0.10 mM	50 mM PB	7.4	AF
Dargel et al. [29] (2019)	25	0.38 ± 0.10 mM	50 mM PB	7.4	AF
Dargel et al. [29] (2019)	30	0.37 ± 0.11 mM	50 mM PB	7.4	AF
Dargel et al. [29] (2019)	35	0.37 ± 0.12 mM	50 mM PB	7.4	AF
Dargel et al. [29] (2019)	40	0.35 ± 0.13 mM	50 mM PB	7.4	AF
Dargel et al. [29] (2019)	45	0.35 ± 0.14 mM	50 mM PB	7.4	AF
Dargel et al. [29] (2019)	50	0.32 ± 0.13 mM	50 mM PB	7.4	AF
Dargel et al. [29] (2019)	23	0.33 ± 0.02 mM	50 mM PB	7.4	T
